# Supplementary material for: AffyMAPSDetector: a software tool to characterize Affymetrix GeneChip™ expression arrays with respect to SNPs
Source: BMC Bioinformatics. 2007 Jul 30;8:276. doi: 10.1186/1471-2105-8-276 (PMC1959249; doi:10.1186/1471-2105-8-276)
Supplement: Additional file 13 — AffyMAPSDetector v1 source code. AffyMAPSDetector source code. [file 1471-2105-8-276-S13.zip › AffyMAPSDetector_v1_src/doc/affychip/snpdetector/model/class-use/GeneInfo.html]

Uses of Class affychip.snpdetector.model.GeneInfo


|  |  |  |  |
| --- | --- | --- | --- |
| **Overview** \| **Package** \| **Class** \| **Use** \| **Tree** \| **Deprecated** \| **Index** \| **Help** | | |  |
| PREV   NEXT | **FRAMES**    **NO FRAMES**     **All Classes** |  |  |


---


## **Uses of Class affychip.snpdetector.model.GeneInfo**

|  |  |
| --- | --- |
| Packages that use GeneInfo | |
| **affychip.snpdetector.model** |  |

Uses of GeneInfo in affychip.snpdetector.model

|  |  |
| --- | --- |
| Methods in affychip.snpdetector.model that return GeneInfo | |
| `GeneInfo` | `SnpDetector.dectectSnpsFor(java.lang.String geneAccessionNumber, java.lang.String locusLinkId)`             THIS METHOD IS USED ONLY FOR EXPERIMENT AND TESTING. |
| `GeneInfo[]` | `DataState.getGeneInfo()` |

|  |  |
| --- | --- |
| Methods in affychip.snpdetector.model with parameters of type GeneInfo | |
| `void` | `SnpDetector.detectSnpsFor(GeneInfo geneInfo)`             This method accesses a gene with a given accession number (from GenBank) and locus-link-id online and searches NCBI snp database for existence of SNPs in this gene's nucleotide-sequence. |
| `void` | `ProbeSet.setGeneInfo(GeneInfo geneInfo)` |
| `void` | `OptimizedSnpDetector.detectSnpsFor(GeneInfo geneInfo)`             This method accesses a gene with a given accession number (from GenBank) and locus-link-id online and searches NCBI snp database for existence of SNPs in this gene's nucleotide-sequence. |
| `void` | `OptimizedSnpDetector.eFetch(java.lang.String refSeqId, GeneInfo geneInfo)`             This method accesses a gene with a given accession number (from GenBank) and locus-link-id online and searches NCBI snp database for existence of SNPs in this gene's nucleotide-sequence. |
| `void` | `DataState.setGeneInfo(GeneInfo[] geneInfo)` |

---


|  |  |  |  |
| --- | --- | --- | --- |
| **Overview** \| **Package** \| **Class** \| **Use** \| **Tree** \| **Deprecated** \| **Index** \| **Help** | | |  |
| PREV   NEXT | **FRAMES**    **NO FRAMES**     **All Classes** |  |  |


---
